# CFTR dysfunction in smooth muscle drives TGFβ dependent airway hyperreactivity

**DOI:** 10.1186/s12931-023-02495-2

**Published:** 2023-08-11

**Authors:** Elizabeth L. Kramer, Kristin M. Hudock, Cynthia R. Davidson, John P. Clancy

**Affiliations:** 1 Department of Pediatrics, University of Cincinnati College of Medicine, Cincinnati, OH USA; 2 Division of Pulmonary Medicine, Cincinnati Children’s Hospital, Cincinnati, OH USA; 3 Division of Adult Pulmonary & Critical Care Medicine, University of Cincinnati, Cincinnati, OH USA; 4 Division of Pulmonary Biology, Cincinnati Children’s Hospital Medical Center, Cincinnati, OH USA; 5 Cystic Fibrosis Foundation, Bethesda, MD USA

**Keywords:** Cystic fibrosis, CFTR, Transforming growth factor beta, Airway smooth muscle, Airway hyperreactivity

## Abstract

**Background:**

The primary underlying defect in cystic fibrosis (CF) is disrupted ion transport in epithelia throughout the body. It is unclear if symptoms such as airway hyperreactivity (AHR) and increased airway smooth muscle (ASM) volume in people with CF are due to inherent abnormalities in smooth muscle or are secondary to epithelial dysfunction. Transforming Growth Factor beta 1 (TGFβ) is an established genetic modifier of CF lung disease and a known driver of abnormal ASM function. Prior studies have demonstrated that CF mice develop greater AHR, goblet cell hyperplasia, and ASM hypertrophy after pulmonary TGFβ exposure. However, the mechanism driving these abnormalities in CF lung disease, specifically the contribution of CFTR loss in ASM, was unknown.

**Methods:**

In this study, mice with smooth muscle-specific loss of CFTR function (*Cftr*^*fl/fl*^; *SM-Cre* mice) were exposed to pulmonary TGFβ. The impact on lung pathology and physiology was investigated through examination of lung mechanics, Western blot analysis, and pulmonary histology.

**Results:**

*Cftr*^*fl/fl*^; *SM-Cre* mice treated with TGFβ demonstrated greater methacholine-induced AHR than control mice. However, *Cftr*^*fl/fl*^; *SM-Cre* mice did not develop increased inflammation, ASM area, or goblet cell hyperplasia relative to controls following TGFβ exposure.

**Conclusions:**

These results demonstrate a direct smooth muscle contribution to CF airway obstruction mediated by TGFβ. Dysfunction in non-epithelial tissues should be considered in the development of CF therapeutics, including potential genetic therapies.

**Supplementary Information:**

The online version contains supplementary material available at 10.1186/s12931-023-02495-2.

## Background

Cystic Fibrosis (CF), a genetic disorder characterized by progressive lung disease, results from mutations in the gene encoding the cystic fibrosis transmembrane conductance regulator (CFTR) protein that disrupt anion transport in epithelia and other tissues throughout the body [1]. Recently approved CFTR modulator therapies increase CFTR function, drastically improving symptom burden and lung function for many people with CF [2]. CFTR dysfunction is not limited to epithelial cells, however. Pathologic alterations in smooth muscle, myeloid cells, endothelial cells, and cartilaginous development have been described in CF [3–8]. These abnormalities may significantly contribute not only to CF disease progression but also to residual disease manifestations after current modulator therapy or future gene therapy.

Airway smooth muscle (ASM) dysfunction is particularly important to understand in the context of CF and plays an important role in symptoms and disease trajectory. Structurally, children with CF have a larger volume of ASM around airways [9]. ASM function is also altered in CF and associated with airway hyperresponsiveness (AHR), which often manifests clinically as wheezing and dyspnea. The majority of people with CF demonstrate significant drops in lung function when challenged with the bronchoconstrictor methacholine, indicating AHR [10]. AHR is particularly worrisome in those with CF. Carrying even a single CFTR mutation is linked to a greater risk of asthma [10]. Clinically, people with CF and AHR also have more rapid loss of lung function and an increased number of pulmonary exacerbations which together may contribute to reduced quality of life and survival [11]. Intrinsic susceptibility to bronchospasm in people with CF may be compounded by commonly used therapies including hypertonic saline and CFTR modulators such as lumacaftor/ivacaftor. Unfortunately, the associated symptoms of chest tightness, cough, and difficulty breathing due to bronchoconstriction may lead to discontinuation of beneficial therapies [12, 13]. Together, these data implicate a defect in CF ASM that leads to worse pulmonary outcomes in people with CF.

ASM function in CF is complex and likely influenced by signaling factors in the pulmonary microenvironment produced by chronic inflammation, infection, prior therapies (e.g. corticosteroid exposure), and/or ASM structure. Previous studies have identified ASM defects but have not clearly demonstrated if ASM-specific CFTR loss is the mechanism driving AHR in vivo [14–17]. Prior studies of human CF ASM in vitro have produced conflicting results regarding CF ASM contractility [6, 15, 16]. Studies of the neonatal CF pig (prior to the onset of infection and inflammation) have shown that loss of CFTR function leads to increased contractility in ASM [14]. These findings point to an inherent CFTR-dependent phenotype in CF ASM. Whether CFTR-independent signaling contributes to this phenotype, however, is unknown. To better understand the mechanism driving increased AHR in people with CF, selective knockout of CFTR in smooth muscle coupled with measurements of lung mechanics in live animal models are necessary.

Previously, we have identified ASM abnormalities in a CF mouse model mediated by TGFβ [18]. Although CF mice do not develop spontaneous lung disease analogous to human CF disease, exposure to TGFβ, a known genetic modifier of CF lung disease, elicits relevant lung disease in CF mice compared with littermate controls [19]. In CF, higher producing TGFβ polymorphisms have been linked to more severe lung disease; furthermore, higher levels of TGFβ in the BAL and plasma are associated with worse outcomes in children with CF [20–22]. Higher producing TGFβ polymorphisms are also associated with worsening asthma severity, highlighting a connection between ASM abnormalities and TGFβ exposure [23]. TGFβ is known to enhance methacholine-induced ASM contraction in vivo via the Smad signaling pathway [24]. The mechanism of TGFβ modification of CF lung disease is unclear and likely multifactorial.

We have previously demonstrated that subacute exposure to physiologic levels of pulmonary TGFβ in F508del CF mice triggers greater abnormalities in lung mechanics, ASM hypertrophy, goblet cell hyperplasia, and methacholine response compared to non-CF mice [18, 19]. The relative contribution of CFTR loss in the ASM versus other tissue types in driving these changes, however, remains a significant knowledge gap. To better understand the role of CFTR specifically in the smooth muscle without the contribution of CFTR dysfunction from other tissue types, we used a smooth muscle-specific knockout mouse in this study.

**Fig. 1:**
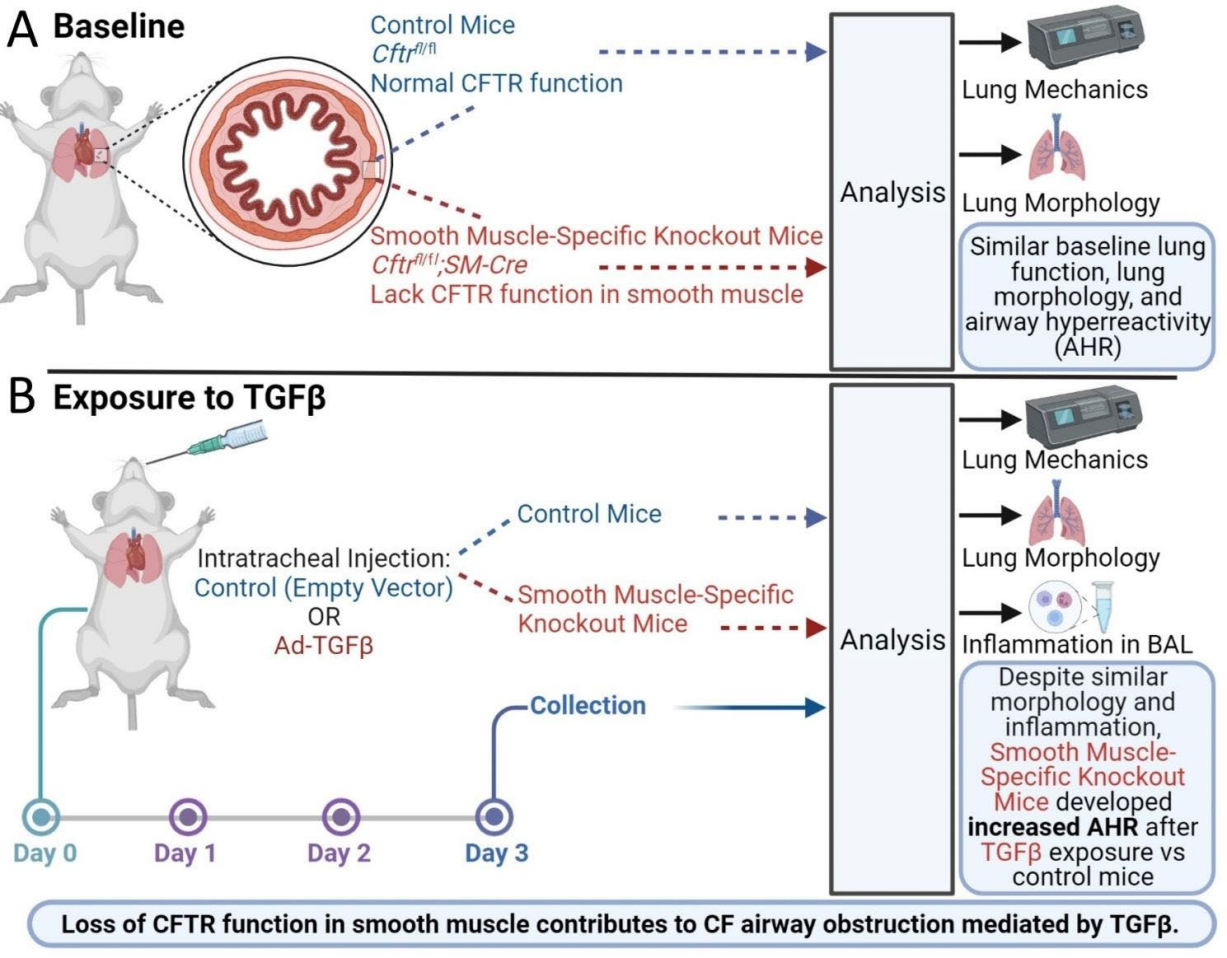
Graphical summary of this study. **A**. At baseline, smooth muscle-specific knockout mice *Cftr*^*fl/fl*^; *SM-Cre* mice) do not demonstrate altered lung mechanics or lung morphology compared to control *Cftr*^*fl/fl*^ mice. **B**. Three days after exposure to Ad-TGFβ or empty vector control, *Cftr*^*fl/fl*^; *SM-Cre* mice had similar pulmonary inflammation and lung morphology (goblet cell hyperplasia and ASM area) compared to control mice. However, only *Cftr*^*fl/fl*^; *SM-Cre* mice developed elevated AHR after TGFβ treatment. This study demonstrates that loss of CFTR function in smooth muscle contributes to CF airway obstruction mediated by TGFβ. Created with BioRender.com

To date, no prior studies have examined the impact of smooth muscle-specific loss of CFTR function on lung physiology using a whole animal model. In this manuscript, we utilized a floxed *Cftr* mouse model to investigate the mechanism driving TGFβ-dependent lung disease in CF, specifically examining the role of CFTR function in smooth muscle and AHR [25]. We examined whether isolated loss of CFTR function in ASM was sufficient to produce AHR in response to TGFβ, independent of the other TGFβ effects on epithelial morphology or inflammation present in F508del mice [19]. We identified increased AHR in mice with CFTR-deficient ASM, without concurrent ASM or goblet cell hyperplasia, indicating an inherent defect in CF ASM as summarized in Fig. 1. Our results highlight a non-epithelial contributor to CF lung disease that is mediated in part by TGFβ and represents a novel target for future therapies. These findings also emphasize how CFTR dysfunction outside of the epithelial compartment can directly contribute to airway obstruction, with relevance to nucleic acid-based therapies in development. Some of the results of these studies have been previously reported in the form of an abstract [26].

## Methods

*Institutional Approval*. This study was approved by the Cincinnati Children’s Hospital Research Foundation’s Institutional Animal Care and Use Committee (IACUC).

*Transgenic Mice*. Conditional CFTR knockout mice (*Cftr*^*fl/fl*^ mice) were obtained from the Case Western Reserve University Cystic Fibrosis Mouse Models Core (Cleveland, OH) [25]. *Cftr*^*fl/fl*^ mice have *loxP* sites flanking exon 10 of *Cftr*, causing loss of CFTR function in the presence of Cre recombinase as previously verified through functional testing [25]. Mice expressing Cre in smooth muscle under the control of the *Sm22* promoter (*SM-Cre* mice; strain #017491) were obtained from The Jackson Laboratory (Bar Harbor, ME). Smooth muscle-specific CFTR loss of function mice (*Cftr*^*fl/fl*^; *SM-Cre* mice) were generated. Non-Cre expressing littermates (*Cftr*^*fl/fl*^ mice) were used as controls.

*Adenoviral Vector*. Ad-TGFβ is a nonreplicating adenoviral vector that contains a TGFβ1 transgene; its pulmonary effects have been previously described [27]. To control for vector effects, an empty adenoviral vector (Ad-dl70-3) was used [28]. Both vectors were delivered at a dose of 5 × 10^7^ pfu. At this dose, the empty vector does not induce lung pathology and is equivalent to PBS intratracheal injection [19]. Adult male and female mice, 4–7 per group, underwent anesthesia with ketamine-xylazine prior to intratracheal administration of Ad-TGFβ (5 × 10^7^ pfu) or empty vector (5 × 10^7^ pfu). Mice were sacrificed at day 3 after administration.

*Lung Mechanics*. After anesthetization with ketamine-xylazine-acepromazine solution, mice were cannulated and measurement of lung mechanics performed with the flexiVent system (SCIREQ, Montreal, Canada). After measurement of baseline lung mechanics, increasing doses of the bronchoconstrictor methacholine (Acetyl-β-methylcholine chloride, Sigma, St. Louis, MO) were administered via nebulization to determine AHR. Pulmonary resistance was measured with twelve forced oscillation perturbations obtained every 12 s.

*Bronchoalveolar Lavage and Cell Counts*. After pulmonary mechanics measurements were completed, bronchoalveolar lavage fluid (BALF) was collected by flushing 1 mL sterile PBS through the tracheal cannula. Total cell count was obtained and Kwik-Diff Stain (Thermofisher Scientific, Ontario, Canada) was performed on cells. Differential cell counts were performed by identifying and counting two hundred cells per mouse.

*Lung Histology and Immunostaining*. After inflation with 10% formalin at a pressure of 25 cm H_2_O, lungs were embedded in paraffin blocks. Staining for tissue structure was performed using Masson’s Trichrome method (Poly Scientific R&D, Bay Shore, NY). Periodic Acid-Schiff (PAS) stain (Poly Scientific R&D, Bay Shore, NY) was performed to stain goblet cells, and airway goblet cell percentage was calculated as previously described [19]. ASM was stained with alpha-smooth muscle actin antibody (αSMA; A2547, Sigma). Morphometric analysis of ASM area corrected to basement membrane (BM) perimeter squared was performed as previously described using MetaMorph software (Molecular Devices, Sunnyvale, CA) [18].

*TGFβ ELISA*. Quantification of active and total TGFβ was measured via ELISA (R + D Systems, Minneapolis, MN) of BALF fluid.

*Western blot analysis*. Whole mouse lungs were collected and homogenized. Analysis of pathways downstream of TGFβ was performed. Primary antibodies used were phosphorylated Smad2 (Ab3849, Millipore, Billerica, MA), Smad2 (CS5339, Cell Signaling, Danvers, MA), phosphorylated Akt Ser 473 (CS4060, Cell Signaling), Akt (CS9272, Cell Signaling), phosphorylated extracellular signal-regulated protein kinases (ERK) 1/2 (CS4370, Cell Signaling), and ERK1/2 (CS9102, Cell Signaling). PhosphoImager (Fujifilm, Valhalla, NY) with MultiGauge software (Fujifilm) was used to quantify protein expression.

*Statistical Analysis*. Statistical analysis was performed with GraphPad Prism (GraphPad Software, San Diego, CA). Comparison between two groups was completed using two-tailed Student’s t-test (for normal distributions) or Mann-Whitney test (for non-normal distributions) as appropriate. Comparisons between three or more groups was performed using one-way ANOVA with Tukey multiple comparisons test. Values are expressed as mean ± standard deviation.

## Results

### ***Cftr***^***fl/fl***^; ***SM-Cre*** mice have normal ASM architecture and function at baseline

To specifically target the knockout of CFTR function in smooth muscle, we utilized a previously developed conditional null *Cftr*^*fl/fl*^ mouse model [25] bred with mice expressing Cre recombinase in smooth muscle (*SM-cre*). *Cftr*^*fl/fl*^ mice have normal CFTR function [25]. Constitutive CFTR null (*Cftr*^*fl/fl*^: *protamine-cre*) mice have been generated and described as phenotypically nearly identical to other CFTR knockout models [25]. However, baseline lung physiology has not been analyzed in *Cftr*^*fl/fl*^; *SM-Cre* mice, which lack CFTR function in all smooth muscle cells. We hypothesized that at baseline, these mice would be phenotypically identical to control littermate *Cftr*^*fl/fl*^ mice without additional stimuli.

**Fig. 2:**
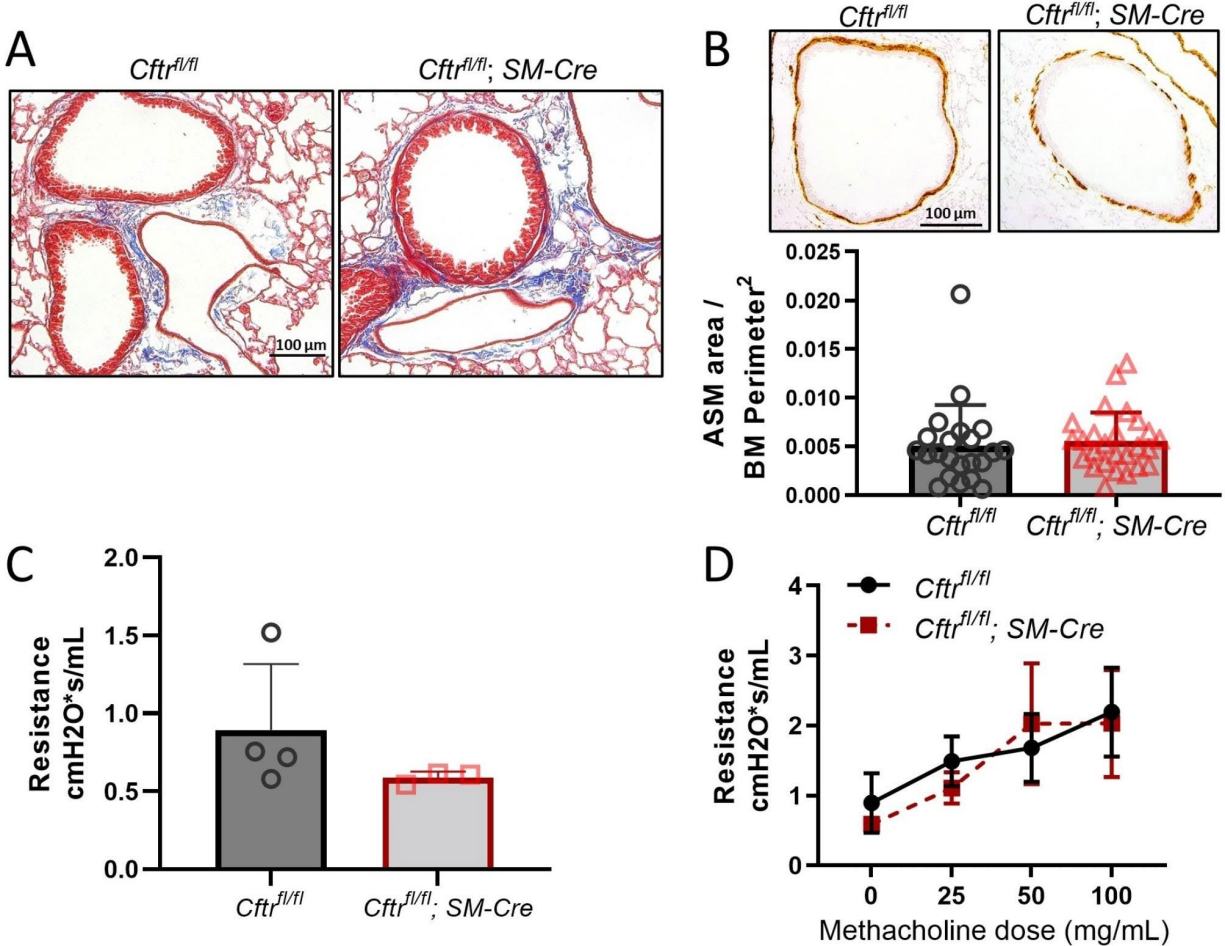
At baseline, smooth muscle-specific CFTR knockout mice (*Cftr*^*fl/fl*^; *SM-Cre* mice) showed no abnormalities in lung morphology or pulmonary resistance. **A**. Trichrome stain of lung sections from *Cftr*^*fl/fl*^; *SM-Cre* mice, which lack CFTR function in smooth muscle, do not reveal abnormalities at baseline compared to control *Cftr*^*fl/fl*^ lung sections. **B**. Staining and morphometric analysis of airway smooth muscle (ASM) with αSMA immunohistochemistry demonstrates similar ASM burden in *Cftr*^*fl/fl*^; *SM-Cre* and control mice. **C**. Baseline lung resistance is unchanged by loss of CFTR function in ASM. **D**. Methacholine challenge of *Cftr*^*fl/fl*^; *SM-Cre* and *Cftr*^*fl/fl*^ mice show no increased airway hyperreactivity (AHR) with loss of CFTR function in smooth muscle. Data are presented as mean ± SD

As we anticipated, *Cftr*^*fl/fl*^; *SM-Cre* mice have unchanged lung architecture compared to control *Cftr*^*fl/fl*^ mice as seen on Trichrome staining (Fig. 2A). Similarly, ASM morphology at baseline is comparable in *Cftr*^*fl/fl*^; *SM-Cre* and *Cftr*^*fl/fl*^ mice, as demonstrated by αSMA staining and morphometric analysis of ASM area (Fig. 2B). Pulmonary resistance is unaffected by loss of CFTR in smooth muscle with no significant changes in baseline resistance, although a nonsignificant trend of lower pulmonary resistance was noted in *Cftr*^*fl/fl*^; *SM-Cre* mice (Fig. 2C). No significant differences in response to methacholine exposure were noted in *Cftr*^*fl/fl*^; *SM-Cre* versus *Cftr*^*fl/fl*^ mice (Fig. 2D).

### TGFβ exposure elicits similar weight loss and inflammation in ***Cftr***^***fl/fl***^ and ***Cftr***^***fl/fl***^; ***SM-Cre*** mice

**Fig. 3:**
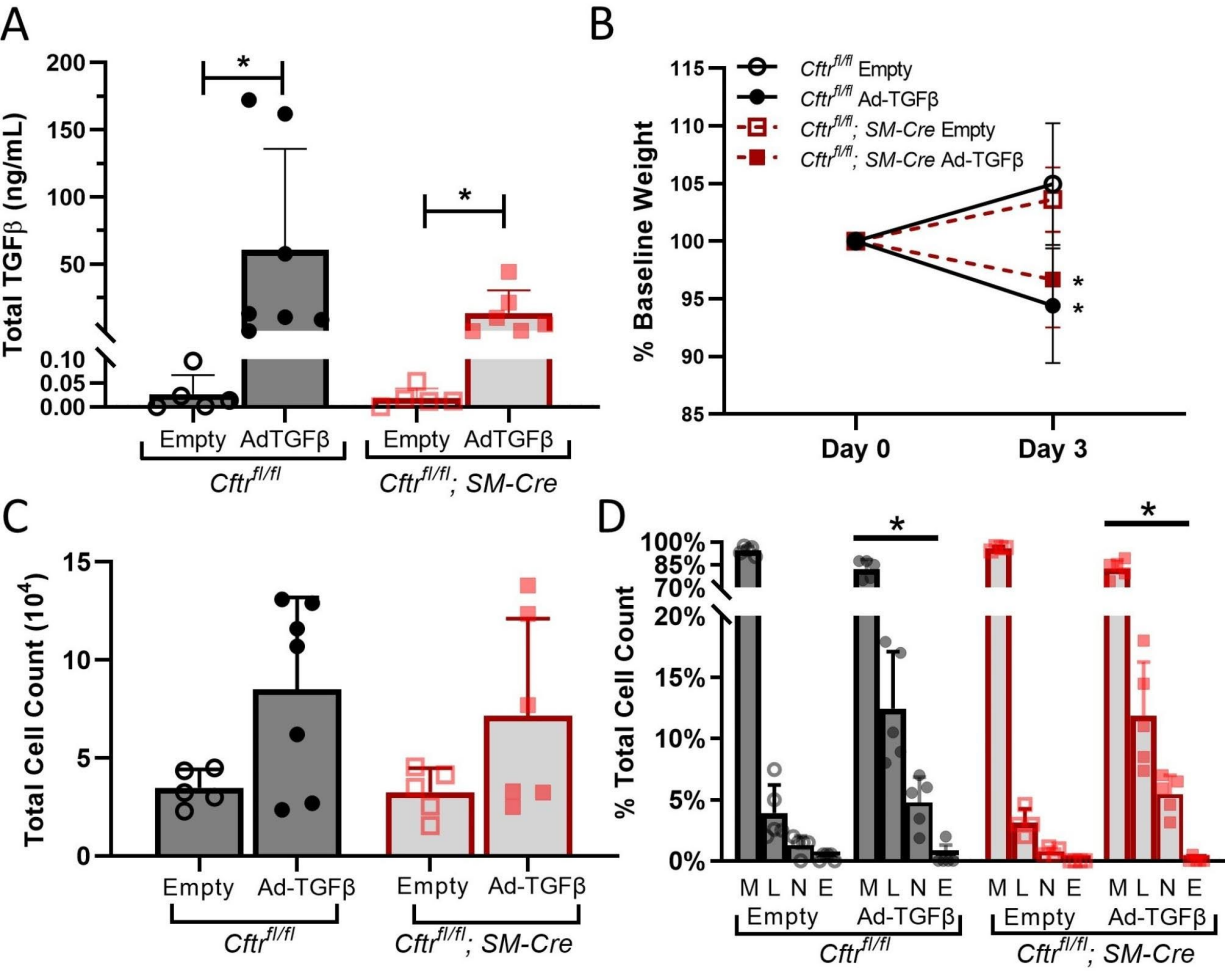
After pulmonary TGFβ exposure, *Cftr*^*fl/fl*^; *SM-Cre* mice demonstrated similar changes in pulmonary TGFβ, weight, and inflammation. **A**. Total TGFβ levels, as determined by ELISA, were significantly increased in BAL fluid three days after pulmonary Adenoviral (Ad)-transforming growth factor (TGF) β exposure. *P < 0.05 by Mann-Whitney test. **B**. Treatment with Ad-TGFβ induced weight loss at day 3 in both control *Cftr*^*fl/fl*^ mice and *Cftr*^*fl/fl*^; *SM-Cre* mice. *P < 0.05 versus respective empty vector control mice of the same genotype by two-tailed t-test. **C**. Both *Cftr*^*fl/fl*^ and *Cftr*^*fl/fl*^; *SM-Cre* mice demonstrated a trend toward increased total BAL cell count after Ad-TGFβ treatment, although this did not reach significance. **D**. Ad-TGFβ treatment caused similar changes in differential cell count of BALF in control *Cftr*^*fl/fl*^ mice and *Cftr*^*fl/fl*^; *SM-Cre* mice, with decreased percentage macrophages and increased percent lymphocytes and neutrophils. *P < 0.05 versus respective empty vector control mice of the same genotype by two-tailed t-test. Data are presented as mean ± SD

Our lab has previously demonstrated that F508del homozygous CF mice demonstrate enhanced TGFβ-induced AHR, bronchodilator response, and goblet cell hyperplasia when compared to littermate controls [18]. To investigate ASM morphology and behavior in the absence of CFTR function in smooth muscle, *Cftr*^*fl/fl*^; *SM-Cre* and control *Cftr*^*fl/fl*^ mice were treated with intratracheal Ad-TGFβ to expose lungs to a physiologically relevant concentration of TGFβ [19]. Control mice were treated with the same dose of intratracheal empty adenoviral vector. In our previous studies in the F508del mouse model, we demonstrated that goblet cell hyperplasia and ASM hypertrophy develop after seven days of TGFβ exposure, while AHR develops after only three days [18, 19]. To explore the role of CFTR dysfunction in driving AHR in the absence of increased mucus secretion and ASM proliferation, a TGFβ exposure time of three days was selected for this study. Three days after intratracheal injection, total TGFβ levels in BAL fluid were significantly increased in Ad-TGFβ treated mice compared to empty vector treated mice in both *Cftr*^*fl/fl*^ and *Cftr*^*fl/fl*^; *SM-Cre* groups (Fig. 3A). As expected, active TGFβ was undetectable in empty vector exposed mice and elevated in both Ad-TGFβ exposed *Cftr*^*fl/fl*^ (3.00 ± 3.96 ng/mL) and *Cftr*^*fl/fl*^; *SM-Cre* (1.00 ± 1.28 ng/mL) mice with no significant difference between TGFβ exposed groups (p = 0.366 by Mann-Whitney test). Ad-TGFβ exposed mice demonstrated greater weight loss as compared to empty vector treated mice of the same genotype (Fig. 3B).

Consistent with our previous studies, Ad-TGFβ exposure provoked pulmonary inflammation [19]. CFTR knockout in ASM did not appear to impact the cellular response to luminal TGFβ. Total cell counts in BAL fluid trended towards an increase in both TGFβ treated groups but did not reach statistical significance (Fig. 3C). Percent macrophages was reduced in both *Cftr*^*fl/fl*^; *SM-Cre* and control *Cftr*^*fl/fl*^ mice after Ad-TGFβ treatment, while percent lymphocytes and neutrophils were significantly increased (Fig. 3D).

### Loss of smooth muscle CFTR function does not impact goblet cell or airway smooth muscle histology after TGFβ exposure

**Fig. 4:**
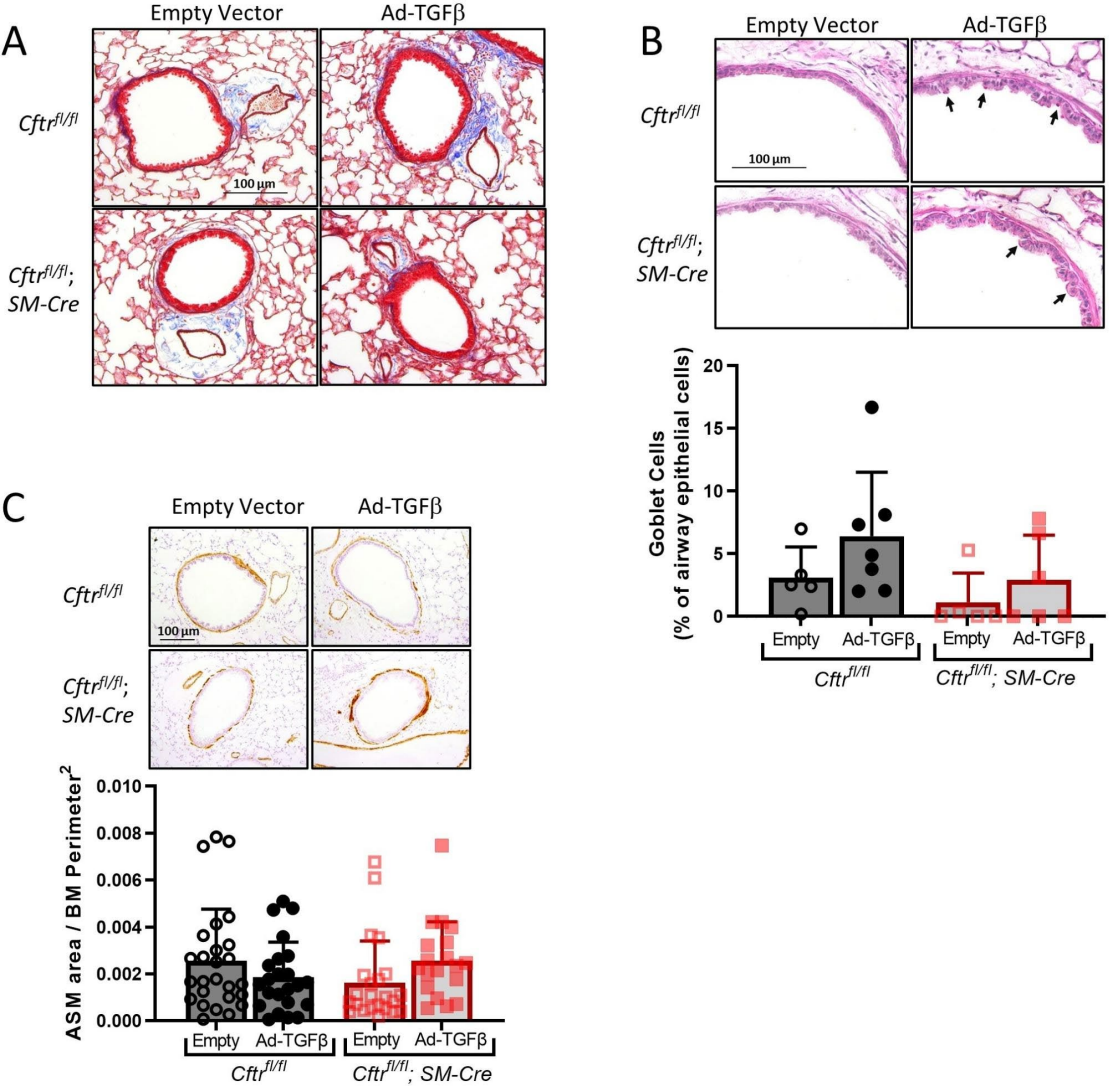
*Cftr*^*fl/fl*^; *SM-Cre* mice treated with TGFβ had no significant goblet cell hyperplasia or alterations in ASM morphology. **A**. Trichrome staining revealed epithelial hyperplasia, increased inflammatory infiltrates around airways, and thickened basement membranes in Ad-TGFβ exposed mice of both genotypes. **B**. Although there was a trend towards increased percentage goblet cells after TGFβ exposure, as determined by morphometric analysis of PAS staining, neither control *Cftr*^*fl/fl*^ mice nor *Cftr*^*fl/fl*^; *SM-Cre* mice demonstrated significant goblet cell hyperplasia. Arrows indicate PAS positive cells. **C**. Morphometric analysis of αSMA stained lung sections showed that Ad-TGFβ exposure did not alter ASM area in either control *Cftr*^*fl/fl*^ or *Cftr*^*fl/fl*^; *SM-Cre* mice. Data are presented as mean ± SD

Lung sections were obtained from Ad-TGFβ and empty vector exposed mice to compare relevant lung histology. Trichrome staining did not reveal significant fibrosis after TGFβ exposure in either group while airway epithelial thickening and patchy inflammatory infiltrates were noted in both *Cftr*^*fl/fl*^ and *Cftr*^*fl/fl*^; *SM-Cre* groups (Fig. 4A). PAS staining was performed to determine if goblet cell hyperplasia was induced by pulmonary Ad-TGFβ, as previously noted in the F508del mouse model after longer TGFβ exposure [18]. Goblet cell counts trended slightly upward in both TGFβ exposed groups, but as expected based upon prior work in the F508del CF mouse model, this did not reach significance (Fig. 4B) [18]. ASM burden was also investigated through immunohistochemistry for αSMA. Morphometric analysis of ASM area demonstrated that neither *Cftr*^*fl/fl*^ nor *Cftr*^*fl/fl*^; *SM-Cre* mice developed significantly more ASM burden three days after Ad-TGFβ exposure, which is consistent with our prior data at this time point (Fig. 4C) [18].

### ***Cftr***^***fl/fl***^; ***SM-Cre*** mice demonstrate increased airway hyperresponsiveness without ASM hyperplasia

**Fig. 5:**
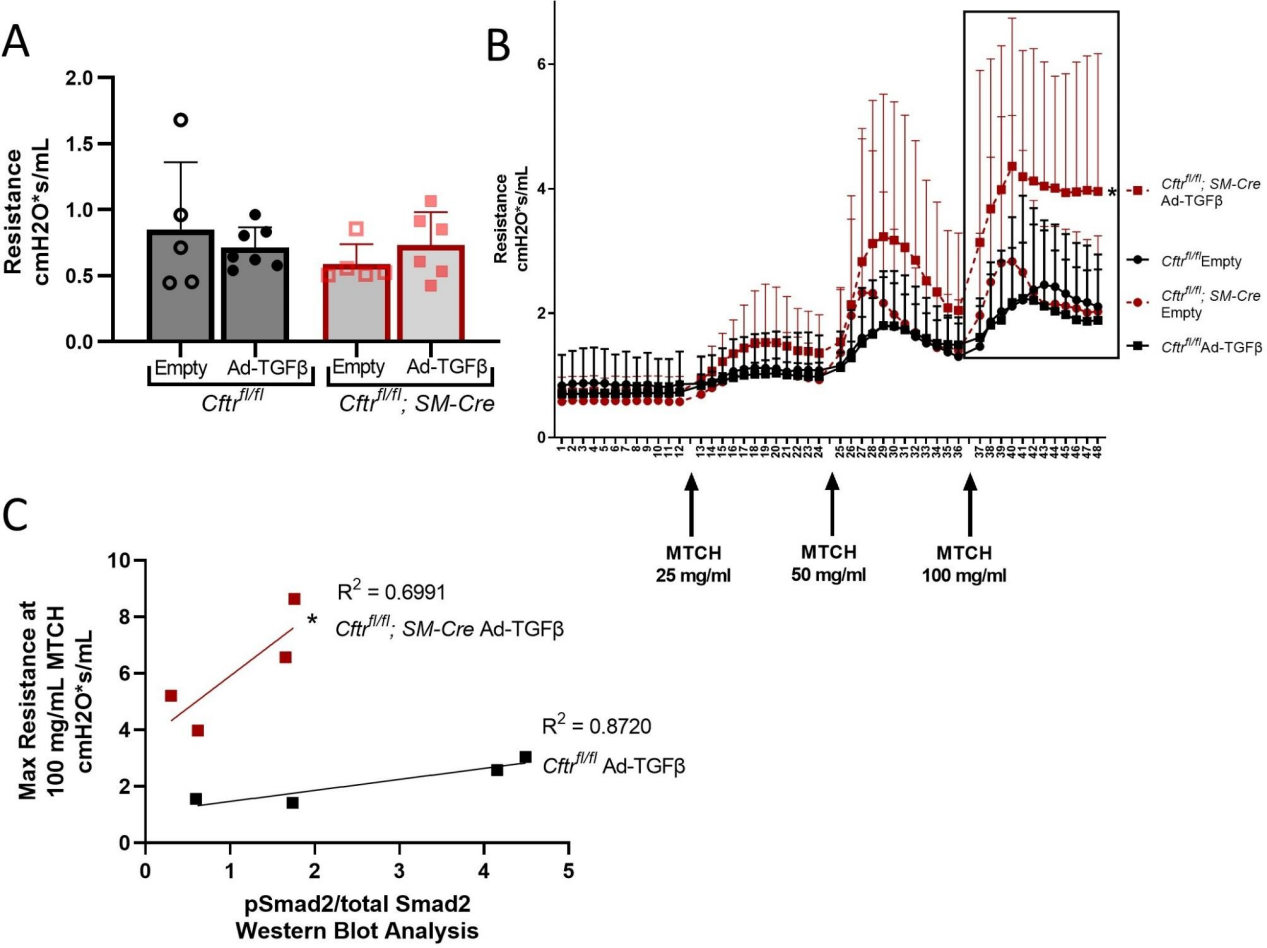
Despite unchanged baseline pulmonary resistance, *Cftr*^*fl/fl*^; *SM-Cre* mice treated with TGFβ had elevated AHR linked to increased Smad signaling. **A**. Baseline pulmonary resistance was unchanged by pulmonary Ad-TGFβ treatment. **B**. In contrast to baseline resistance, *Cftr*^*fl/fl*^; *SM-Cre* mice had increased AHR after pulmonary TGFβ exposure. Methacholine challenge testing showed increased AHR in Ad-TGFβ exposed *Cftr*^*fl/fl*^; *SM-Cre* mice compared to Ad-TGFβ exposed control *Cftr*^*fl/fl*^ mice. Box indicates maximal lung resistance values used for linear regression analysis in 4C. *P < 0.05 for area under the curve after 50 mg/mL and 100 mg/mL doses of nebulized methacholine vs Ad-TGFβ exposed control *Cftr*^*fl/fl*^ mice by one-way ANOVA with Tukey’s post hoc analysis. **C**. Linear regression analysis demonstrated a relationship between increased Smad2 signaling, as measured via Western blot analysis, and maximal pulmonary resistance in both *Cftr*^*fl/fl*^ and *Cftr*^*fl/fl*^; *SM-Cre* mice after exposure to pulmonary Ad-TGFβ. Regression line comparison showed a significantly higher y-intercept in *Cftr*^*fl/fl*^; *SM-Cre* mice, indicating an increased resistance at any given level of Smad2 signaling in the absence of CFTR smooth muscle function. *P < 0.05 versus Ad-TGFβ exposed control *Cftr*^*fl/fl*^ mice by ANCOVA analysis. Data are presented as mean ± SD

Previously, we have demonstrated that pulmonary TGFβ provokes AHR without ASM hypertrophy in the F508del CF mouse model [18]; however, it was unclear if this was due to inherent abnormalities in CF ASM or secondary effects produced by absent CFTR function in the overlying airway epithelium. In the current study, baseline lung resistance was not impacted by Ad-TGFβ treatment in either genotype (Fig. 5A). Despite similar ASM architecture and baseline resistance, TGFβ treatment produced significant increases in pulmonary resistance in only the *Cftr*^*fl/fl*^; *SM-Cre* mice following 50 and 100 ng/mL doses of nebulized methacholine (Fig. 5B). The results indicate that TGFβ-dependent increases in AHR were specific to mice lacking CFTR function in the ASM.

We further hypothesized that TGFβ signaling pathway activation would have a greater correlation with lung resistance in *Cftr*^*fl/fl*^; *SM-Cre* mice lacking CFTR function in ASM. Smad2 signaling, determined by Western blot analysis of phospho-Smad2 corrected to total Smad2, was quantified in whole lung homogenates to assess canonical intracellular signaling downstream of TGFβ. Non-canonical PI3K signaling (indicated by phospho-Akt corrected to total Akt) and MAPK signaling (phospho-ERK1/2 corrected to total ERK1/2) were also measured. TGFβ treatment increased pSmad2/total Smad2 signaling in control *Cftr*^*fl/fl*^ mice; while a trend towards increased Smad signaling was seen in *Cftr*^*fl/fl*^; *SM-Cre* mice, this did not reach significance (P = 0.1) (Additional File 1: Figure S1). As whole lung homogenates were used in the Western blot analysis, it is likely that Smad signaling in Ad-TGFβ exposed tissues, primarily the bronchioles and alveoli [27], is underestimated. Non-canonical PI3K and ERK pathways were unchanged in both groups (Additional File 1: Figure S1).

To determine the relationship between canonical Smad signaling and bronchoconstriction, phospho-Smad2/total Smad2 detection was compared to maximal lung resistance after stimulation of bronchoconstriction with high dose (100 mg/mL) methacholine (see box, Fig. 5B). Comparison of regression lines for each TGFβ-exposed mouse group showed that while the slopes were not significantly different (p = 0.085), the Y intercept for the *Cftr*^*fl/fl*^; *SM-Cre* group was significantly higher than control *Cftr*^*fl/fl*^ mice (Fig. 5C; p = 0.0068). This indicates that mice lacking CFTR in smooth muscle demonstrated significantly higher methacholine-induced pulmonary resistance associated with any given level of TGFβ signaling pathway activation.

## Discussion

Although prior studies have supported the concept that CF is associated with ASM defects, this study is the first to identify and quantify ASM-specific effects of CFTR dysfunction on pulmonary function in a whole animal model. The novel mouse model studied in this paper utilizes the Cre-lox system to eliminate CFTR function only in smooth muscle cells. This contrasts with our prior studies of TGFβ-mediated effects on CF lung disease [18, 19] in the F508del CF mouse model lacking CFTR function in all pulmonary tissues. Use of this smooth muscle-specific CFTR knockout model allows clearer assignment of observed physiologic changes produced by TGFβ to a primary CFTR defect in ASM (see summary Fig. 1). We first established that, at baseline, mice lacking CFTR function in ASM were similar to littermate controls (Fig. 2) and have similar phenotypic, histological, and inflammatory responses to TGFβ exposure compared with littermate controls expressing smooth muscle CFTR (Figs. 3 and 4). After provocation with TGFβ, mice with CFTR-deficient ASM had increased methacholine-induced AHR when compared to controls (Fig. 5). There was no concurrent ASM or goblet cell hypertrophy. As goblet cells and pulmonary inflammation are similar in both *Cftr*^*fl/fl*^; *SM-Cre* and control mice, TGFβ-induced AHR in the knockout mice cannot be attributed to increased inflammation or mucus burden. Our results suggest that dysfunctional CFTR in smooth muscle drives increased airway resistance and obstruction, which is consistent with prior studies in both the CF pig model and in people with CF treated with ivacaftor [14, 29]. This inherent abnormality in CF ASM function highlights an understudied role of CFTR that may have important clinical implications.

TGFβ is an established genetic modifier of CF lung disease, with higher producing polymorphisms linked to more severe CF lung disease [20]. In addition, TGFβ is increased with CF pulmonary exacerbations and *Pseudomonas aeruginosa* infection [21, 22]. People with higher producing TGFβ polymorphisms have an increased risk of developing asthma, demonstrating the relevance of TGFβ in driving AHR in pulmonary disease beyond CF [23]. In studies of isolated human airways, TGFβ increased both basal and methacholine-induced contraction through the Smad pathway [24]. TGFβ enhanced ASM excitation-contraction coupling pathways, thereby driving increased ASM contraction and AHR [24]. These results are consistent with our prior work demonstrating increased AHR in CF mice after exposure to pulmonary TGFβ [18]. Thus, TGFβ exposure is a clinically relevant stimuli that contributes to several pathologic features of CF lung disease including airway obstruction.

In the era of highly effective CFTR modulators, many individuals with CF have extended life expectancy. Understanding the complex pathophysiology resulting from CFTR dysfunction will be critical to provide focused clinical care and identify residual symptoms. ASM abnormalities in people with CF are clinically relevant and may result in airway obstruction and symptoms including dyspnea and cough that detract from quality of life [9, 10]. AHR in people with CF has also been linked to loss of lung function and increased pulmonary exacerbation rates, important sources of morbidity among patients with CF [10]. Certain medical therapies (CFTR modulator lumacaftor-ivacaftor, hypertonic saline, and inhaled antibiotics) may be especially difficulty to tolerate in patients prone to AHR due to bronchospasm-induced cough and chest tightness; in some cases, AHR may preclude patients from experiencing the full benefits of these therapies [12, 13]. CFTR localizes to the sarcoplasmic reticulum (SR) and loss of function results in prolonged cytosolic calcium release, suggesting that reduced anion transport through CFTR impairs SR calcium reuptake, causing prolonged and dysfunctional contraction of CF ASM [14, 30]. Previous studies have identified CF ASM defects but have not clearly demonstrated if ASM-specific CFTR loss is the mechanism driving AHR in vivo [14–17]. Thus, our work to elucidate the ASM-specific effects of CFTR dysfunction in an animal model is critical to understand the nature of airway obstruction and improve clinical care for people with CF.

Although highly effective CFTR modulator therapy improves clinical outcomes for many people with CF, definitive evidence is not yet available regarding the specific effects of CFTR modulator therapy on ASM in CF. Evidence from precision cut porcine lung slices shows that ivacaftor pretreatment attenuates methacholine-induced airway narrowing [14]. Ivacaftor has been shown to quickly improve lung function and air trapping within 48 h in people with CF; it is unclear if this is due to smooth muscle effects, restored mucociliary clearance, or both mechanisms [29]. Even if CFTR modulators do lessen ASM dysfunction in CF, ongoing inflammation and infection occur even with modulator therapy, which may also drive continuing AHR despite modulator use [31, 32]. Case studies have described how interruption of modulator therapy can provoke a rapid decline in lung health [33]. If modulators do improve CF ASM dysfunction, abrupt cessation of a modulator may trigger bronchospasm in susceptible people. A better understanding of the impact of modulators on CF ASM will be important to monitor and treat the effects of interruptions in modulator therapy. Ultimately, more research is needed to determine the impact of CFTR modulators on ASM and AHR and to identify the effects of modulator withdrawal.

Defining tissue-specific contributions of CFTR dysfunction to CF disease is of growing importance as CFTR restorative therapies advance to new and younger CF populations and as the degree of effectiveness of modulators across various tissues is unknown. The relative role each tissue type plays in driving symptoms may differ, or even evolve, in the context of CFTR modulator therapy. In contrast to our prior results in the F508del CF mouse model, *Cftr*^*fl/fl*^; *SM-Cre* mice do not demonstrate goblet cell hyperplasia after TGFβ treatment [18]. These findings support the notion that more extreme goblet cell hyperplasia in the CF lung is driven by non-smooth muscle effects of CFTR dysfunction, potentially including epithelial and inflammatory cells. Studies of isolated human CF epithelial cells have not found an inherent tendency toward excessive goblet cell hyperplasia, indicating this may be influenced by multiple cell types in the complex CF pulmonary environment [34]. Tissue-specific knockout models may help to determine which cell types contribute to specific pathologies in CF. Understanding the relative role of each tissue will be crucial as gene therapy for CF advances. This knowledge may be key to developing targeted therapies and providing these when patients are most likely to benefit.

This study has limitations. CF mouse models do not develop the spontaneous, mucopurulent and progressive lung disease historically seen in people with CF [35–37]. In this regard, they may be a useful model for defining pulmonary abnormalities that persist in the absence of mucus plugging or overwhelming infection, as would be the case for many individuals with CF who are treated with highly effective modulators. In this study, use of murine models has allowed us to identify unique relationships between CFTR expression in different cell and organ compartments. The *Cftr*^*fl/fl*^; *SM-Cre* mouse demonstrates Cre expression in all smooth muscle types, not solely ASM. It is possible, therefore, that there are other smooth muscle pathologies in *Cftr*^*fl/fl*^; *SM-Cre* mice, such as vascular and intestinal smooth muscle dysfunction; abnormalities in these types of smooth muscle would be unlikely to contribute to AHR. Our study also does not rule out the possibility that loss of CFTR function in other non-ASM tissues also contributes to ASM dysfunction. Prior studies have demonstrated that both epithelial and smooth muscle CFTR loss contribute to the bowel obstruction phenotype in CF mice [38, 39]. A similar combined effect of the epithelium and ASM may be necessary to provoke the full ASM phenotype.

Further studies are needed to better understand the role of TGFβ and ASM dysfunction in CF lung disease. Loss of CFTR function in non-ASM tissue types, such as the pulmonary epithelium, may also drive AHR and airway obstruction; a conditional epithelial CFTR knockout model would allow this to be tested. Longer TGFβ exposure will be necessary to determine if loss of smooth muscle CFTR is associated with ASM hypertrophy or goblet cell hyperplasia, as noted after seven days of TGFβ exposure in the F508del mouse model [19]. Infection in the CF lung drives loss of lung function [40], and pulmonary LPS exposure in a CFTR knockout mouse model induced more extreme lung remodeling and increased pulmonary resistance compared to control mice [41]. The effect of LPS and infection on lung function and remodeling in this smooth muscle-specific CFTR knockout model is a direction for future study.

## Conclusions

In summary, the results of our studies clarify an ASM-specific defect in CF that is directly attributable to loss of CFTR function which requires TGFβ to fully manifest. They also highlight the power of organ-specific expression to define disease mechanisms. Strategic use of advanced animal models in which CFTR function can be systematically eliminated in specific tissues may help clarify the benefits and limitations of new CF therapies and identify tissues that should be targeted with gene therapy or other therapeutic agents.

### Electronic supplementary material

Below is the link to the electronic supplementary material.

Supplementary Material 1

## Data Availability

The datasets used and analyzed during the current study are available from the corresponding author on reasonable request.

## Abbreviations

AHR: Airway Hyperreactivity
ASM: Airway Smooth Muscle
CF: Cystic Fibrosis
CFTR: Cystic Fibrosis Transmembrane Conductance Regulator
TGFβ: Transforming Growth Factor β1

## References

[1] Rowe SM, Miller S, Sorscher EJ (2005). Cystic fibrosis. N Engl J Med.

[2] Collins FS (2019). Realizing the dream of molecularly targeted therapies for cystic fibrosis. N Engl J Med.

[3] Bonfield TL, Hodges CA, Cotton CU, Drumm ML (2012). Absence of the cystic fibrosis transmembrane regulator (cftr) from myeloid-derived cells slows resolution of inflammation and infection. J Leukoc Biol.

[4] Bonvin E, Le Rouzic P, Bernaudin JF, Cottart CH, Vandebrouck C, Crie A (2008). Congenital tracheal malformation in cystic fibrosis transmembrane conductance regulator-deficient mice. J Physiol.

[5] Meyerholz DK, Stoltz DA, Namati E, Ramachandran S, Pezzulo AA, Smith AR (2010). Loss of cystic fibrosis transmembrane conductance regulator function produces abnormalities in tracheal development in neonatal pigs and young children. Am J Respir Crit Care Med.

[6] Michoud MC, Robert R, Hassan M, Moynihan B, Haston C, Govindaraju V (2009). Role of the cystic fibrosis transmembrane conductance channel in human airway smooth muscle. Am J Respir Cell Mol Biol.

[7] Robert R, Norez C, Becq F (2005). Disruption of CFTR chloride channel alters mechanical properties and cAMP-dependent Cl- transport of mouse aortic smooth muscle cells. J Physiol.

[8] Declercq M, Treps L, Carmeliet P, Witters P (2019). The role of endothelial cells in cystic fibrosis. J Cyst Fibros.

[9] Regamey N, Ochs M, Hilliard TN, Muhlfeld C, Cornish N, Fleming L (2008). Increased airway smooth muscle mass in children with asthma, cystic fibrosis, and non-cystic fibrosis bronchiectasis. Am J Respir Crit Care Med.

[10] Eggleston PA, Rosenstein BJ, Stackhouse CM, Alexander MF (1988). Airway hyperreactivity in cystic fibrosis. Clinical correlates and possible effects on the course of the disease. Chest.

[11] Nielsen AO, Qayum S, Bouchelouche PN, Laursen LC, Dahl R, Dahl M (2016). Risk of asthma in heterozygous carriers for cystic fibrosis: a meta-analysis. J Cyst Fibros.

[12] Elkins MR, Robinson M, Rose BR, Harbour C, Moriarty CP, Marks GB (2006). A controlled trial of long-term inhaled hypertonic saline in patients with cystic fibrosis. N Engl J Med.

[13] Wainwright CE, Elborn JS, Ramsey BW (2015). Lumacaftor-Ivacaftor in patients with cystic fibrosis homozygous for Phe508del CFTR. N Engl J Med.

[14] Cook DP, Rector MV, Bouzek DC, Michalski AS, Gansemer ND, Reznikov LR (2016). Cystic fibrosis transmembrane Conductance Regulator in Sarcoplasmic Reticulum of Airway smooth muscle. Implications for Airway Contractility. Am J Respir Crit Care Med.

[15] Matusovsky OS, Kachmar L, Ijpma G, Panariti A, Benedetti A, Martin JG (2019). Contractile Properties of Intrapulmonary Airway smooth muscle in cystic fibrosis. Am J Respir Cell Mol Biol.

[16] Jang JH, Panariti A, O’Sullivan MJ, Pyrch M, Wong C, Lauzon AM (2019). Characterization of cystic fibrosis airway smooth muscle cell proliferative and contractile activities. Am J Physiol Lung Cell Mol Physiol.

[17] Govindaraju V, Michoud MC, Ferraro P, Arkinson J, Safka K, Valderrama-Carvajal H (2008). The effects of interleukin-8 on airway smooth muscle contraction in cystic fibrosis. Respir Res.

[18] Kramer EL, Madala SK, Hudock KM, Davidson C, Clancy JP (2020). Subacute TGFbeta exposure drives Airway Hyperresponsiveness in cystic fibrosis mice through the PI3K pathway. Am J Respir Cell Mol Biol.

[19] Kramer EL, Hardie WD, Madala SK, Davidson C, Clancy JP (2018). Subacute TGFbeta expression drives inflammation, goblet cell hyperplasia, and pulmonary function abnormalities in mice with effects dependent on CFTR function. Am J Physiol Lung Cell Mol Physiol.

[20] Drumm ML, Konstan MW, Schluchter MD, Handler A, Pace R, Zou F (2005). Genetic modifiers of lung disease in cystic fibrosis. N Engl J Med.

[21] Harris WT, Muhlebach MS, Oster RA, Knowles MR, Noah TL (2009). Transforming growth factor-beta(1) in bronchoalveolar lavage fluid from children with cystic fibrosis. Pediatr Pulmonol.

[22] Harris WT, Muhlebach MS, Oster RA, Knowles MR, Clancy JP, Noah TL (2011). Plasma TGF-β_1_ in pediatric cystic fibrosis: potential biomarker of lung disease and response to therapy. Pediatr Pulmonol.

[23] Pulleyn LJ, Newton R, Adcock IM, Barnes PJ (2001). TGFbeta1 allele association with asthma severity. Hum Genet.

[24] Ojiaku CA, Cao G, Zhu W, Yoo EJ, Shumyatcher M, Himes BE et al. TGF-beta1 evokes human airway smooth muscle cell shortening and hyperresponsiveness via Smad3. Am J Respir Cell Mol Biol. 2017. 10.1165/rcmb.2017-0247OC PMC5946330 28984468

[25] Hodges CA, Cotton CU, Palmert MR, Drumm ML (2008). Generation of a conditional null allele for cftr in mice. Genesis.

[26] Kramer E, Hudock K, Davidson C, Clancy J (2022). Smooth muscle-specific cystic fibrosis transmembrane conductance regulator loss induces airway hyperreactivity in response to transforming growth factor beta. J Cyst Fibros.

[27] Warshamana GS, Pociask DA, Fisher KJ, Liu JY, Sime PJ, Brody AR (2002). Titration of non-replicating adenovirus as a vector for transducing active TGF-beta1 gene expression causing inflammation and fibrogenesis in the lungs of C57BL/6 mice. Int J Exp Pathol.

[28] Bett AJ, Haddara W, Prevec L, Graham FL (1994). An efficient and flexible system for construction of adenovirus vectors with insertions or deletions in early regions 1 and 3. Proc Natl Acad Sci U S A.

[29] Adam RJ, Hisert KB, Dodd JD, Grogan B, Launspach JL, Barnes JK (2016). Acute administration of ivacaftor to people with cystic fibrosis and a G551D-CFTR mutation reveals smooth muscle abnormalities. JCI Insight.

[30] Cook DP, Adam RJ, Zarei K, Deonovic B, Stroik MR, Gansemer ND (2017). CF airway smooth muscle transcriptome reveals a role for PYK2. JCI Insight.

[31] Hisert KB, Heltshe SL, Pope C, Jorth P, Wu X, Edwards RM (2017). Restoring cystic Fibrosis Transmembrane Conductance Regulator function reduces airway Bacteria and inflammation in people with cystic fibrosis and chronic lung infections. Am J Respir Crit Care Med.

[32] Harris JK, Wagner BD, Zemanick ET, Robertson CE, Stevens MJ, Heltshe SL (2020). Changes in Airway Microbiome and inflammation with Ivacaftor Treatment in patients with cystic fibrosis and the G551D mutation. Ann Am Thorac Soc.

[33] Trimble AT, Donaldson SH (2018). Ivacaftor withdrawal syndrome in cystic fibrosis patients with the G551D mutation. J Cyst Fibros.

[34] Adam D, Roux-Delrieu J, Luczka E, Bonnomet A, Lesage J, Merol JC (2015). Cystic fibrosis airway epithelium remodelling: involvement of inflammation. J Pathol.

[35] Davidson DJ, Rolfe M (2001). Mouse models of cystic fibrosis. Trends Genet.

[36] Zeiher BG, Eichwald E, Zabner J, Smith JJ, Puga AP, McCray PB (1995). A mouse model for the delta F508 allele of cystic fibrosis. J Clin Invest.

[37] Darrah RJ, Bederman IR, Mitchell AL, Hodges CA, Campanaro CK, Drumm ML (2013). Ventilatory pattern and energy expenditure are altered in cystic fibrosis mice. J Cyst Fibros.

[38] Hodges CA, Grady BR, Mishra K, Cotton CU, Drumm ML (2011). Cystic fibrosis growth retardation is not correlated with loss of cftr in the intestinal epithelium. Am J Physiol Gastrointest Liver Physiol.

[39] Vitko MS (2016). Intestinal dysfunction in cystic fibrosis.

[40] Gibson RL, Burns JL, Ramsey BW (2003). Pathophysiology and management of pulmonary infections in cystic fibrosis. Am J Respir Crit Care Med.

[41] Bruscia EM, Zhang PX, Barone C, Scholte BJ, Homer R, Krause DS (2016). Increased susceptibility of Cftr-/- mice to LPS-induced lung remodeling. Am J Physiol Lung Cell Mol Physiol.

