# Supplementary material for: CFTR dysfunction in smooth muscle drives TGFβ dependent airway hyperreactivity
Source: Respir Res. 2023 Aug 11;24:198. doi: 10.1186/s12931-023-02495-2 (PMC10416378; doi:10.1186/s12931-023-02495-2)
Supplement: Supplementary file 1 — Supplementary Material 1 [file 12931_2023_2495_MOESM1_ESM.pdf]

Supplemental Figure S1

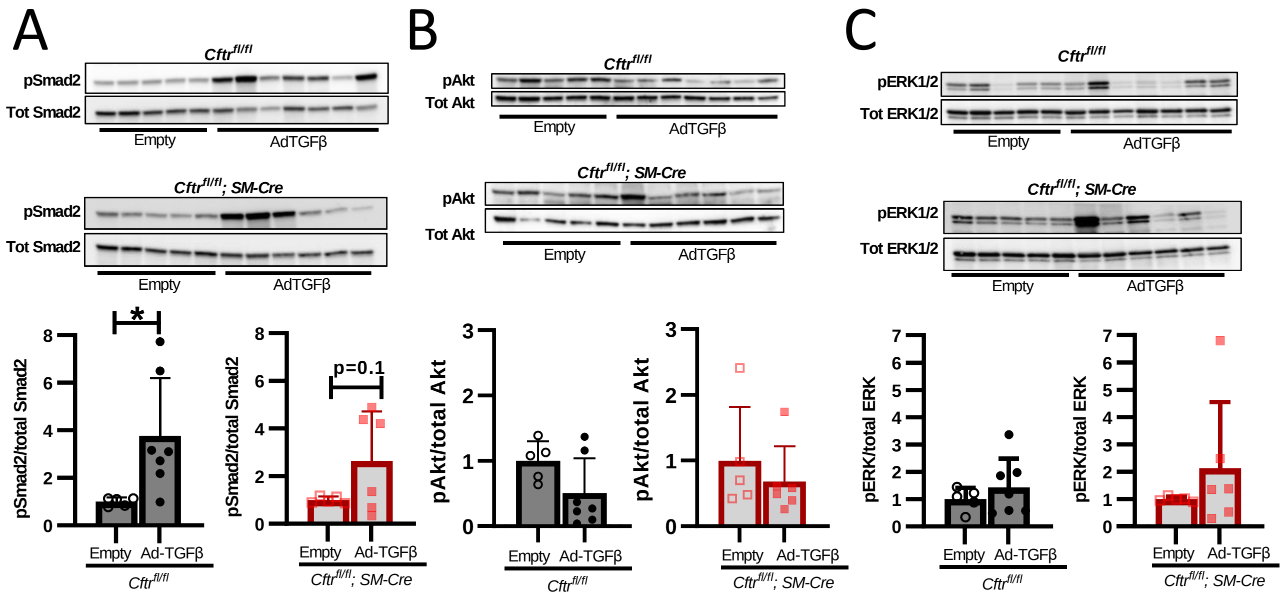

Fig S1. Western blot analysis of whole lung homogenates indicates increases in canonical (Smad2) signaling and no significant changes in non-canonical (PI3K and MAPK) signaling after pulmonary Ad-TGFβ treatment. **A**. Significantly increased phospho-Smad2 normalized to total Smad2 was seen in control *Cftr^fl/fl^* mice exposed to Ad-TGFβ as compared with empty vector treated mice, with *Cftr^fl/fl^*; *SM-Cre* mice showing a trend towards increased Smad signaling. * $P < 0.05$ by two-tailed t-test. **B**. No significant differences in TGFβ-induced PI3K signaling, as measured by phospho/total Akt, were noted. **C.** Similarly, ERK1/2 (MAPK) signaling was not significantly altered by TGFβ exposure in either mouse group. Data are presented as mean ± SD.
